# Supplementary material for: Neutrophil-to-Lymphocyte Ratio and Early Variation of NLR to Predict In-Hospital Mortality and Severity in ED Patients with SARS-CoV-2 Infection
Source: J Clin Med. 2021 Jun 9;10(12):2563. doi: 10.3390/jcm10122563 (PMC8229672; doi:10.3390/jcm10122563)
Supplement: Supplementary file 1 [file jcm-10-02563-s001.zip › jcm-1189117-supplementary.pdf]

Tables and Figures

Figure 1: Flowchart of the study

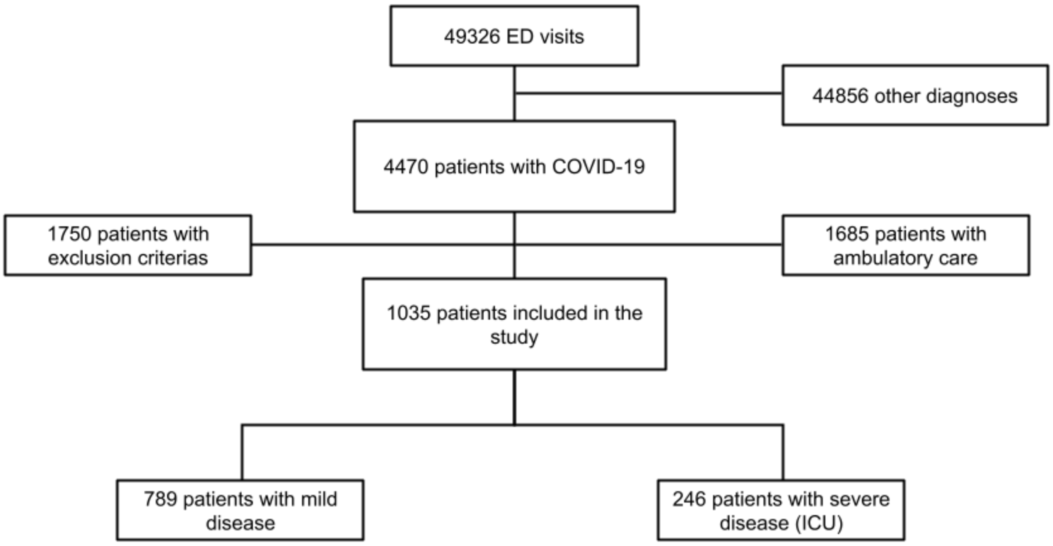

Abbreviations: ED: Emergency Department, ICU: intensive care unit

Table 1: Demographic, baseline and laboratory characteristics of patients with COVID-19

	All patients N=1035	Moderate N=789	Severe N=246	p
**General characteristics**				
Age (years)	69 [58-79]	70 [58-81]	66 [57.3-72]	<0.001*
Gender male	609 (58.8)	433 (54.9)	176 (71.5)	<0.001*
Obesity (BMI ≥30)	281 (36.9)	193 (35.0)	88 (41.9)	0.076
**Chronic medical illness**				
Hypertension	587 (56.7)	453 (57.4)	134 (54.5)	0.416
Diabetes mellitus	275 (26.7)	202 (25.6)	73 (26.6)	0.207
CKD	237 (23.2)	199 (25.5)	38 (15.8)	0.002*
Cardiovascular illness	357 (34.5)	291 (36.9)	66 (26.8)	0.004*
Total Autonomy	796 (77.2)	569 (72.4)	227 (92.7)	<0.001*
Respiratory illness	203 (19.6)	151 (19.1)	52 (21.1)	0.490
**Laboratory findings**				
CRP (mg/L)	81 [39-142.3]	68 [33-128]	124 [76-192]	<0.001*
Lymphocyte ($\times 10^9$/L)	870 [630-1200]	900 [640-1220]	780 [590-1122]	0.003*
Lymphocyte H24 ($\times 10^9$/L)	940 [670-1300]	1010 [710-1360]	800 [570-1110]	<0.001*
Neutrophil ($\times 10^9$/L)	4930 [3430- 6932]	4730 [3370-6620]	5510 [3760-8160]	<0.001*
Neutrophil H24 ($\times 10^9$/L)	4680 [3300-6765]	4395 [3005-6175]	6010 [4130-8210]	<0.001*
Admission NLR	5.4 [3.5-9.3]	5.2 [3.2-8.7]	6.6 [4.1-11.1]	<0.001*
H-24 NLR	5 [3.1-8.2]	4.4 [2.7-7]	7.4 [4.7-12.5]	<0.001*
ΔNLR >0 (%)	334 (41.3)	211 (35.3)	123 (58.0)	<0.001*
Δ NLR	-0.64 [-2.88-1.29]	-0.88 [-3.09-0.79]	0.48 [-1.78-3.72]	<0.001*
**Outcome**				
Hospital stay (days)	10 [7-17.3]	8 [6-12]	24 [17-38]	<0.001*
Intra-hospital mortality	139 (13.6)	82 (10.4)	57 (24.1)	<0.001*

Data are all expressed in median [Q1–Q3] n (%) where n is the total number of patients with available data. * $p < 0.05$.

Abbreviations: BMI = body mass index, CKD= Chronic kidney disease, ED= Emergency Department, O2= oxygen, °C=Celsius degree, CRP=C reactive protein, NLR= neutrophil to lymphocyte ratio, ΔNLR = difference between NLR (NLR H-24 - NLR at admission).

Table 2: Biochemichal factors associated with severe COVID-19 (admission to the ICU)

				Multivariate analysis**	
	**All**	**Moderate**	**Severe**	OR (95%CI)	p-value
CRP	81 [39-142.3]	68 [33-128]	124 [76- 92]	1.007 [1.005-1.010]	<0.001*
Admission lymphocytes	870 [630-1200]	900[640-1220]	780[590-1122.5]	1000 [1.000-1.000]	0.841
Admission NLR	5.4 [3.5-9.3]	5.2 [3.2-8.7]	6.6 [4.1-11.1]	0.971 [0.940-1.004]	0.082
H-24 NLR	5 [3.1-8.2]	4.4 [2.7-7.0]	7.4 [4.7-12.5]	1.117 [1.060-1.176]	<0.001*
ΔNLR >0 (%)	334 (41.3)	211 (35.3)	123 (58.0)	1.877 [1.160-3.036]	0.010 *

Data are all expressed in median [Q1–Q3] or n (%) where n is the total number of patients with available data. * p < 0.05, ** model adjusted on gender, Body mass index, C reactive protein, Creatinine, Admission NLR, NLR H24 and ΔNLR.

Abbreviations: OR= odds ratio, BMI = body mass index, CRP=C reactive protein, NLR= neutrophil to lymphocyte ratio, ΔNLR = difference between NLR (NLR H-24 - NLR at admission).

<u>Figure 2</u>: Receiver operating characteristics (ROC) curve for NLR as a predictive factor of severe COVID-19

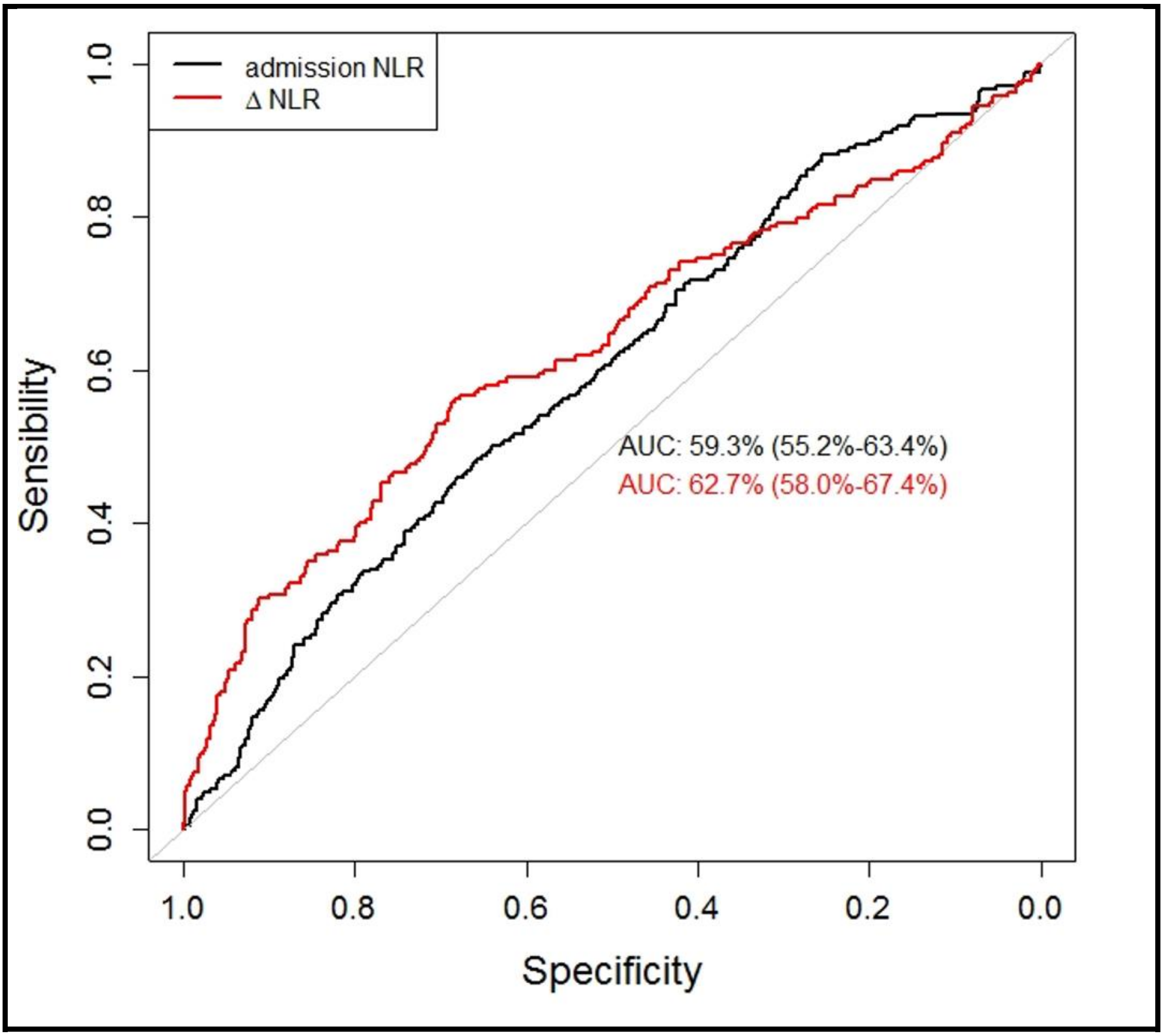

To predict critical illness	Area under curve	Cut-off	Sensibility	Specificity	Multivariate analyse with cut-off OR (95%CI)	p
Admission NLR	0.593 [0.552-0.634]	6.883	48.3 [41,9-54,8]	65.7 [62.2-69.0]	0.975 [0.947-1.005]	0.097
ΔNLR	0.627 [0.580-0.674]	0.222	56.1 [49.2-62.9]	68,3 [64.4-72.1]	2.187 [1.348-3.547]	0.002*

Data are all expressed in median [Q1-Q3], *: p<0.005, **: model adjusted on Gender, Body mass index, C reactive protein, Creatinine, Admission NLR, NLR H24 and Δ NLR.

<u>Abbreviations</u>: OR= odds ratio, NLR: neutrophil lymphocyte ratio, ΔNLR = difference between NLR (NLR H-24 - NLR at admission).

Table 3: Biochemichal and predictive factors of mortality in COVID-19 patients

			Univariate analysis		Multivariate analysis**	
	Survivors	Non-survivors	OR (95% CI)	p-value	OR (95% CI)	p-value
CRP	100.0 [56-158]	78.5 [37-139]	1.003 [1.001-1.005]	0.006*	1.002 [0.999-1.005]	0.276
Admission lymphocytes	720 [500-1000]	890[650-1120	0.999 [0.999-1]	0.004	1.000 [0.999-1.001]	0.967
Admission NLR	7.6 [4.3-11.8]	5.2 [3.3-8.8]	1.023 [1.006-1.042]	0.010*	1.020 [0.987-1.055]	0.232
H-24 NLR	7.9 [5.1-14.1]	4.6[2.9-7.5]	1.059 [1.035-1.084]	<0.001*	1.006 [0.963-1.050]	0.798
Δ NLR >0 (%)	271 (39.0)	58 (55.2)	1.931 [1.277-2.920]	0.002*	2.142 [1.132-4.056]	0.019*

Data are all expressed in median [Q1–Q3] or n (%) where n is the total number of patients with available data. * $p < 0.05$, ** model adjusted on Gender, Body mass index, C reactive protein, Creatinine, Admission NLR, NLR H24 and Δ NLR.

Abbreviations: OR= odds ratio, BMI = body mass index, CRP=C reactive protein, NLR= neutrophil to lymphocyte ratio, ΔNLR = difference between NLR (NLR H-24 - NLR at admission).

Figure 3: Receiver operating characteristics (ROC) curve for NLR as a predictive factor of mortality in COVID-19 patients

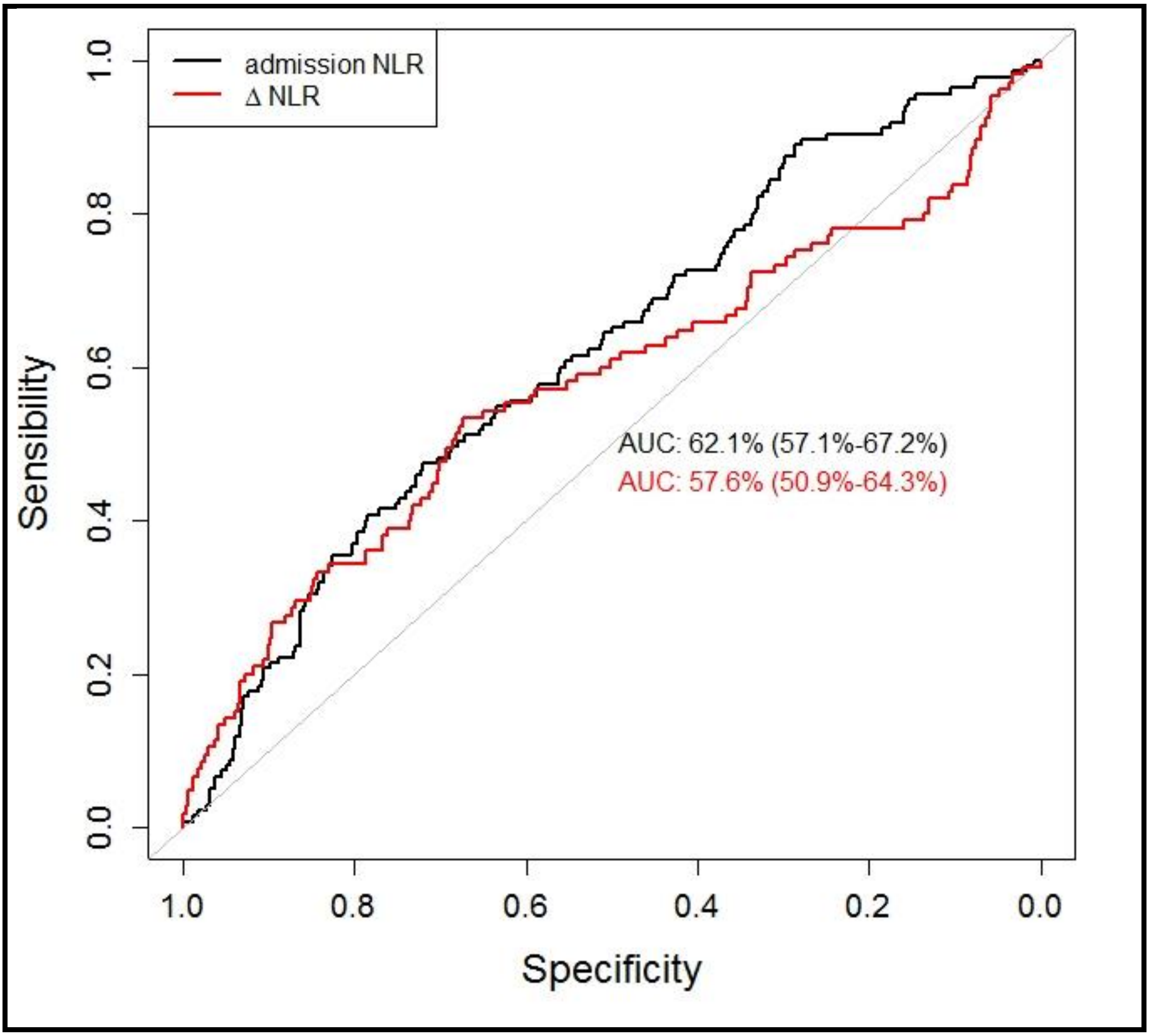

To predict mortality	Area under curve	Cut-off	Sensibility	Specificity	Multivariate analysis with cut-off**	
					OR (95%CI)	p
Admission NLR	0,621 [0,571-0,672]	8.236	47.4 [38,8-56,2]	71.9 [68,8-74,9]	1.025 (0.989-1.063)	0.176
ΔNLR	0,576 [0,509-0,643]	0.411	53.3 [43,3-63,1]	67.3 [63,7-70,8]	2.71 (1.404-5.245)	0.003*

Data are all expressed in median [Q1–Q3], * p<0.005, **: model adjusted on Gender, Body mass index, C reactive protein, Creatinine, Admission NLR, NLR H24 and ΔNLR.

Abbreviations: OR= odds ratio, NLR= neutrophil lymphocyte ratio, ΔNLR = difference between NLR (NLR H-24 - NLR at admission).
